# Engineered Cell Membrane‐Derived Nanoparticles in Immune Modulation

**DOI:** 10.1002/advs.202102330

**Published:** 2021-10-24

**Authors:** Yixiao Yang, Kai Wang, Yuanwei Pan, Lang Rao, Gaoxing Luo

**Affiliations:** ^1^ Institute of Burn Research The First Affiliated Hospital State Key Lab of Trauma Burn and Combined Injury Chongqing Key Laboratory for Disease Proteomics Third Military Medical University (Army Medical University) Chongqing 400038 China; ^2^ Key Laboratory of Medical Molecular Virology (MOE/NHC/CAMS) School of Basic Medical Sciences and Shanghai Public Health Clinical Center Shanghai Medical College Fudan University Shanghai 200032 China; ^3^ Institute of Biomedical Health Technology and Engineering Shenzhen Bay Laboratory Shenzhen 518132 China

**Keywords:** cell membranes, engineering strategies, immunomodulation, nanoparticles

## Abstract

Immune modulation is one of the most effective approaches in the therapy of complex diseases, including public health emergency. However, most immune therapeutics such as drugs, vaccines, and cellular therapy suffer from the limitations of poor efficacy and adverse side effects. Fortunately, cell membrane‐derived nanoparticles (CMDNs) have superior compatibility with other therapeutics and offer new opportunities to push the limits of current treatments in immune modulation. As the interface between cells and outer surroundings, cell membrane contains components which instruct intercellular communication and the plasticity of cytomembrane has significantly potentiated CMDNs to leverage our immune system. Therefore, cell membranes employed in immunomodulatory CMDNs have gradually shifted from natural to engineered. In this review, unique properties of immunomodulatory CMDNs and engineering strategies of emerging CMDNs for immune modulation, with an emphasis on the design logic are summarized. Further, this review points out some pressing problems to be solved during clinical translation and put forward some suggestions on the prospect of immunoregulatory CMDNs. It is anticipated that this review can provide new insights on the design of immunoregulatory CMDNs and expand their potentiation in the precise control of the dysregulated immune system.

## Graphical abstract

Enabled by recent advances in materials science and nanotechnology, emerging cell membrane‐derived nanoparticles hold great potential to provide better solutions for immune modulation. By reviewing the state‐of‐the‐art cell membrane‐derived nanoparticles and highlighting the promising technology development, this review intends to spark further research and development activity in this critical research area.

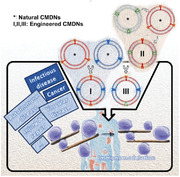

## 1 Introduction

Throughout the coronavirus health crisis, from health officials to individuals, we are all deeply aware of the significance of vaccines.^[^ ^1^ ^]^ Since the famous experiments conducted in 1796 by Edward Jenner that led to the creation of the first smallpox vaccine, our human beings have made numerous attempts to fight against diseases by leveraging our immune system.^[^ ^2^ ^]^ Vaccines, both preventive and therapeutic, are one of multiple representative approaches to modulate our immune system by artificially providing nondisease‐causing antigens.^[^ ^3^ ^]^ Generally, antigens can be administered in various forms according to characteristics of the pathogenic microorganisms. For example, inactivated or killed vaccines are an approach to fight against infections by whole, inactivated reagents. Such vaccines demand for high requirements on production safety, for instance, biosafety level 3 production workshops.^[^ ^4^ ^]^ Specific components from pathogenic microbes are also highly immunogenic, which is termed as subunit vaccines. Polysaccharide vaccines such as meningococcal vaccines are physically purified from bacterial capsules. Prophylactic human papillomavirus (HPV) vaccines are prepared from virus‐like particles (VLPs) which produced by engineered L1 protein self‐assembles to form empty shells that resemble HPV VLPs.^[^ ^5^ ^]^ Nuclear acid‐based vaccine is a new approach to vaccine, which is composed of antigen‐encoding DNA or mRNA, usually formulated in liposomes.^[^ ^6^ ^]^ If continuous monitoring for problems and side effects certify the security, the nuclear acid‐based vaccines will become a type of mainstream vaccines in the near future.

The scope of immune modulation is far beyond activating specific immune responses, and the role of our immune system is more than defending the invasion of foreign invaders like bacteria and viruses. It has been verified that immune disorders have mingled with the initiation and progression of many diseases.^[^ ^7^ ^]^ For example, patients suffering from viral pneumonia (such as SARS‐Cov‐2; COVID‐19) often accompanied by cytokine storm and autoantibodies, the uncontrolled immune responses lead to multi‐organ failure and even death.^[^ ^8^ ^]^ Monoclonal antibody therapy targeted to neutralizing the main inflammatory cytokines or to block the virus’ attachment and entry into human cells was shown in clinical trials to reduce COVID‐19‐related hospitalization.^[^ ^1^, ^9^ ^]^ Moreover, immunological components in tumor microenvironment are educated to be incompetent and submit to tumor growth and metastasis.^[^ ^10^ ^]^ In order to alleviate diseases by rebalancing the dysregulated immune system, many immune regulation strategies have been developed according to their distinguishing features.^[^ ^11^ ^]^ Unlike the conventional vaccines, for instance, there is another potent vaccine termed "dendritic cell vaccine (DC vaccine)" which is a tumor antigens‐loaded dendritic cell‐mediated cellular therapy employed to arousing the exhausted immune components.^[^ ^12^ ^]^ As the most efficient antigen‐presenting cells (APCs), DCs provide the arranged tumor antigens to the downstream effector cells, triggering immune surveillance against tumor cells. Apart from DC vaccines awaken cancer‐fighting components with equipped APCs in vitro, chimeric antigen receptor T‐cell immunotherapy (CAR‐T) are approaches boosting immune response directly by manufacturing T cells to find and destroy cancer cells. These cell‐based immunostimulating therapies restore the body's immune defense to cancer. In addition to vaccines, antibodies, and immune effect cellular therapy, lots of drugs are developed as immunomodulators. Some of them target to block immune checkpoint, such as PD‐1 and PD‐L1, exhibiting remarkable clinical responses in some cases.^[^ ^13^ ^]^ Although the continued evolution of immune therapy has created better solutions for dealing with some of the most pressing hurdles in clinic, the current therapeutics are still limited by factors such as lack of potency or serious adverse effect.

Recent advances in nanotechnology and materials science have offered many new opportunities to push the limit of current therapeutics. Among these promising approaches, cell membrane‐derived nanoparticles (CMDNs), which possessed superiority in drug delivery, have attracted much attention.^[^ ^14^ ^]^ The most distinguishing feature of CMDNs is their inherent biocompatibility.^[^ ^15^ ^]^ Rapid clearance by the immune system is the major hurdle of the in vivo biomedical applications. After disguised by the cell membrane, the nanoparticles would be perceived as the source cell and have a long circulation life in the body. For instance, CD47, an important component in "don't eat me" signaling cascade which tells the phagocytes do not eat these CD47 expressing cells.^[^ ^16^ ^]^ In dealing with cases which seek longer circulation time for immune regulating reagents, membrane from erythrocytes or platelets could serve as eligible candidates.^[^ ^15^, ^17^ ^]^

Next, CMDNs possess targeting effect inherited from parent membrane.^[^ ^18^ ^]^ The interaction between adhesive molecules and their ligands forms the molecular base of the targeting specificity. Almost all leukocytes, platelets, and mesenchymal stem cells (MSCs) express massive adhesive molecules, exhibiting substantial affinity to the endothelium of inflammation site.^[^ ^19^ ^]^ As such, a large amount of effort has been put to make nanoparticles to selectively accumulate in the inflamed tissues via assembling together with cell membrane.^[^ ^20^ ^]^ It should be noted that an expansion of the adhesion‐related molecules on the cytomembrane will be triggered once these cells are activated.^[^ ^21^ ^]^ Therefore, stimulation of the candidate cells before membrane extraction can often achieve better results in the fabrication of CMDNs. Other than adhesive molecules‐based functionalization, homogeneity of membrane is also a cause that led to the targeting effect.^[^ ^22^ ^]^ For example, CMDNs from cancer cells show enhanced tumor accumulation partially due to the homogeneity.^[^ ^23^ ^]^ In practical application, both the above features could be employed in the design of target‐orientation CMDNs.

In terms of immune modulation, benefits of employing CMDNs are far more than in drug delivery. Instead of creating new solutions from the ground‐up, immune regulatory CMDNs often take design cues directly from existing approaches for immune modulation. This review mainly focus on the recent advances of CMDNs in immune modulation, with an emphasis on the design logic. We started with the properties of cell membrane employed in immune modulation, analyzing the molecular basis of immune intervention and the corresponding biomedical applications. Next, the roles of cell membrane‐derived nanovesicles in immune regulation are summarized. Some commonly used components loaded within CMDNs are discussed. On the basis of the former analysis, we introduced engineering strategies of CMDNs in immune regulation. Via analyzing some representative advances of immune regulatory CMDNs, the engineering strategies and design logic were sketched. The final section covers some critical problems to be solved before clinical transformation and gives perspectives in immune regulation.

## 2 Properties of Cell Membrane Employed in Immunoregulatory CMDNs

**Figure 1:**
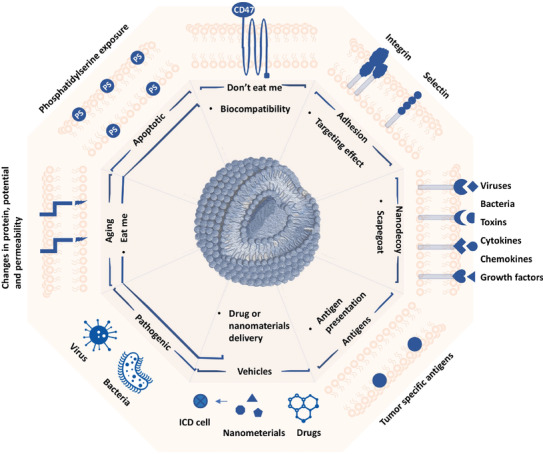
Properties of cell membrane employed in CMDNs. Components on cell membrane encode cues for intercellular communication. Superiority of CMDNs, including long half‐times, targeted effect, vaccine, and nanodecoy, rely on the molecular basis of cytomembrane. CD47, an essential member of “don't eat me” signal, makes CMDNs of long circulation time. Multiple adhesion molecules, such as integrin and selectin, instruct CMDNs’ aggregation to the inflammation or tumor site. Specific antigens on tumor cell membrane provide a facile access for personalized vaccination. Apoptotic cells, aging cells, and pathogenic microbes elicit “eat me” signal mainly through components on cell membrane or outer surface of pathogens. Also, receptors on membrane turn CMDNs into scapegoat to adsorb corresponding ligands, including viruses, bacteria, toxins, cytokines, chemokines, and growth factors.

As the interface between cells and the microenvironment, components expressing on cell membrane instruct behavior of the cell as well as external responses to the cell.^[^ ^24^ ^]^ Cell membrane itself preserves most of the information to communicate with other cells, mainly depending on components embedded on the bilayer structure (**Figure** **1**). As mentioned above, CD47 encodes a message about "don't eat me." And adhesive molecular and homogeneity of cell membrane determines the targeted property of CMDNs. Similarly, communicating with components of our immune system in a language they are familiar with will undoubtedly make the immune modulation easier. Hence, making the interaction principle clear between cells and our immune system will help us to develop better immunoregulatory CMDNs. In this section, we will discuss properties of cell membrane employed in immunoregulatory CMDNs and their biomedical applications.

In contrast to "don't eat me" signal, there are also components trigger "eat me" responses for the phagocytes in circulation, which provides an excellent access for immune regulation by CMDNs (Figure 1). Especially macrophages and DCs, they are phagocytes as well as APCs.^[^ ^25^ ^]^ This property is extremely useful in vaccine design.^[^ ^3^, ^12^, ^26^ ^]^ As an example, derivations from pathogenic microbes, such as simple sugars, glycans, peptides, or proteins, can cause phagocytosis of monocyte–phagocyte system.^[^ ^27^ ^]^ In addition, marked by alterations in protein, potential, and permeability, aging cells are usually cleaned up by macrophages in the body.^[^ ^28^ ^]^ By taking inspiration from nature, introducing specific antigens by imitating pathogenic microbes or senescence cells are good alternatives for immunoregulatory CMDNs.^[^ ^29^ ^]^ Furthermore, it is feasible to expose antigen epitopes by directly engineered CMDNs as cell‐free APCs. Recent studies have shown that mice immunized with engineered CMDNs have successful elicited desirable immune responses in tumor prevention and therapy. Overall, CMDNs‐based vaccine is one of the most popular and most critical application in immune modulation.^[^ ^30^ ^]^ Instead of merely depending on the innate function of the natural membranes, post‐processing of natural membrane is usually necessary for specific antigen presentation in CMDNs fabrication. Meanwhile, the versatile engineering strategies also are of great interpretation of the advantages of the synthetic platforms.

A collection of biological processes requires receptor–ligand interaction. Meanwhile, many ligands, including cytokines, chemokines, growth factors, bacterial toxins, viruses, etc., also play critical roles in immune dysregulation and disease progression.^[^ ^31^ ^]^ A significant portion of receptors is embedded in cell membranes, as a result, all kinds of cytomembranes potentiate to work as nanodecoy (Figure 1).^[^ ^32^ ^]^ As a prime example, CD4^+^ T cell mimicking CMDNs could absorb human immunodeficiency virus (HIVs) for acquired immunodeficiency syndrome (AIDS) remission.^[^ ^33^ ^]^ In the same manner, mosquito medium host cell membrane‐based nanoparticles effectively absorb Zika Virus (ZIKV) and inhibit ZIKV replication in ZIKV‐susceptible cells.^[^ ^34^ ^]^ The fact of the matter is to find out the pivotal ligands leading to aggravation of the condition, their target cells, and the specific receptors. In case membrane‐sourcing cells are difficult to obtain, it is also alternative to exogenously express the receptors on common membranes by genetic engineering.

## 3 Roles of Cell Membrane Nanovesicles in Immune Modulation

**Figure 2:**
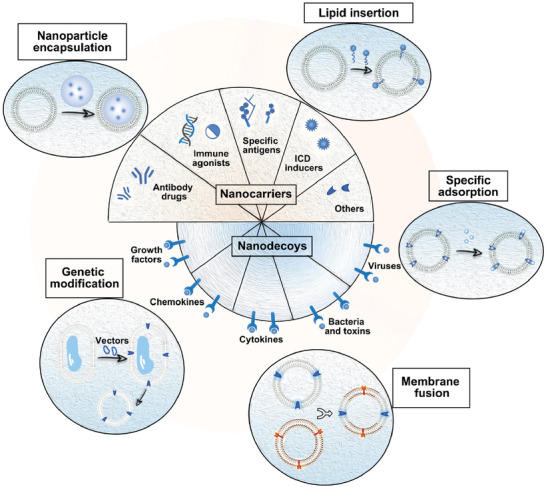
Engineering strategies of CMDNs in immune modulation. In general, cell membrane plays a role of vehicle in immune regulation, either carrying regulation‐related reagents as nanocarriers, or removing harmful components as nanodecoys. Multiple immunomodulatory reagents could be employed in the platform, including antibody drugs, immune agonists, specific antigens, ICD inducers, et al. These components can be loaded within the core of nanovesicles, as well as embedded on the outer surface of cell membrane. When used as nanodecoys, it mainly depends on the receptors on cell membrane to adsorb their corresponding ligands. Moreover, five basic engineering strategies of CMDNs are illustrated, in addition to the most common encapsulation, there are also lipid insertion, specific adsorption, membrane fusion, and genetic modification.

As natural analog of liposomes, the phospholipid bilayer structure of cell membranes endows CMDNs with the inherent function as nanocarriers. The most explored application for CMDNs is drug delivery, attributing to the compatibility of cell membrane and the hollow structure of cell membrane vesicles. While less investigated, CMDNs as nanodecoys also represent an identical application. Both nanocarriers and nanodecoys are vehicle‐based, of which the former is to supplement and the latter is to deplete (**Figure** **2**).

### 3.1 Nanocarriers

Using CMDNs to aid in delivery has become a common strategy. In terms of immune modulation, the regulatory function of CMDNs is largely dependent on the components cell membrane loaded. Regarding the pathological status of patients’ immune system, it is of great importance to select symptomatic immunomodulatory reagents and deliver them in an efficient way. Various immunomodulatory reagents have been explored in the process and we roughly divide them into the following categories.

#### 3.1.1 Antibody Drugs

The work of antibody is a notable factor in immune system and antibody drugs are common approaches to treat certain types of cancers, infectious, or autoimmune diseases. Immune components in tumor microenvironment, for instance, are usually incapable and the tumor‐associated tolerance in turn promotes tumor progression. A lot of neutralizing antibody drugs have been explored both in the lab and in the clinic. Some immune agonist antibody like programmed cell death protein 1 (PD1) and cytotoxic T lymphocyte 4 (CTLA4) blockades are identical monoclonal antibody drugs which are usually employed in the design of CMDNs to rescue the suppressed immune defense.^[^ ^35^ ^]^ In another example, many anti‐SARS‐Cov2 antibodies to block the binding of virus and the host cells have been authorized for emergency use by Food and Drug Administration (FDA) up to now.^[^ ^9^ ^]^ In addition, some bispecific antibody drugs reported in recent studies are used to attach both the cancer cells and specific immune cells together to elicit enhanced immune responses against cancer cells. Combined with cell membrane‐mediated biomimetic delivery strategies, antibody drugs are able to advance their functionality and get rid of some unwanted side‐effects. For instance, Gao et al.’s work indicated that erythrocyte membrane‐coated anti‐hTERT mAb showed longer circulation time and more efficient tumor accumulation than free mAb.^[^ ^36^ ^]^ In another example, Li et al. prepared cluster of differentiation 64 (CD64), a natural catcher of the fragment crystalline (Fc) of monomeric immunoglobulin G (IgG), and over‐expressed it on the cell membrane nanovesicles (NVs) as PD‐L1 antibody delivery vehicle (CD64‐NVs‐aPD‐L1), which elicited significantly enhanced CD‐8+ T cell proliferation and tumor growth inhibition compared with free aPD‐L1.^[^ ^37^ ^]^

#### 3.1.2 Immune Agonists

Immune agonists are reagents which are used to elicit enhanced immune responses. Most of these reagents, such as polysaccharides or DNAs, are structurally resemble pathogenic microbes and are recognized by pattern recognition receptors (PRRs).^[^ ^38^ ^]^ One area toward which researchers have placed much attention and effort is Toll‐like receptors (TLRs) and each TLR has its microbial sourcing ligands.^[^ ^39^ ^]^ One notable example is CpG‐oligodeoxynucleotides (CpG‐ODNs), a short synthetic DNA containing unmethylated CpG motifs which are disguised as bacterial DNA and is often employed as adjuvant to stimulate innate immune cells bearing TLR9 and Th1 cells.^[^ ^40^ ^]^ Therapeutic vaccines against cancer equipped with CpG‐ODNs often elicit boosted antigen presentation and the induction of antigen specific cellular and humoral responses. Besides TLRs, there are still some other PRRs, of which researchers have shown particular interest is stimulator of interferon gene (STING).^[^ ^41^ ^]^ Progressively, novel small molecule STING agonists with favorable drug properties are being developed.^[^ ^42^ ^]^

#### 3.1.3 Specific Antigens

The CMDNs‐loaded specific antigens as vaccines against bacteria, viruses, or cancer is one most energetic area in recent studies toward immune regulatory CMDNs. Over the past 200 years, numerous vaccines have been explored. Taking design inspiration from conventional vaccines as well as the most recently studied cellular therapy, engineered CMDNs have potentiate the safety and efficiency of current vaccination paradigm.^[^ ^14^, ^43^ ^]^ As analog of subunit vaccines, cell membrane vesicles embedded with specific antigens also elicit equal and often better vaccination potency.^[^ ^32^, ^44^ ^]^ Moreover, majority of microbe or tumor‐specific antigens just expressed on membrane, which provide a prime access for natural membrane vesicles as vaccines. One such example, outer membrane vesicles (OMVs) generated from Gram‐negative bacteria contain components which could initiate innate and subsequent adaptive immune responses.^[^ ^45^ ^]^ As such, OMVs and their derivatives are frequently applied in CMDNs‐based vaccines. In mice lethal dose challenge study, all OMVs vaccines‐immunized mice survived for 10 days, whereas all the sham‐immunized mice died within 24 h.^[^ ^46^ ^]^ In particular, with regard to complex diseases like cancer, tumor‐specific antigens or tumor‐associated antigens show diversity and individual characteristics. It can be hard to replicate all‐round antigens artificially while tumor cell membranes from patients could provide the whole array of and personalized antigens effortlessly.^[^ ^47^ ^]^ Thus, tumor cell membrane‐mediated CMDNs will bring about an encouraging evolution in cancer therapy.

#### 3.1.4 Immunogenic Cell Death (ICD) Inducers

Many drugs or biomaterials themselves are not initially used to regulate immunity, however, our immune system is boosted by the process of these drugs work. ICD caused by inducers is featured by the exposure of damage‐associated molecular patterns and subsequently recruit corresponding APCs to deal with the threatening cells. Many chemodrugs and photodynamic reagents are prime inducers to cause ICD.^[^ ^48^ ^]^ As an example, a myeloid‐derived suppressor cell (MDSC) membrane‐coated iron oxide magnetic nanoparticle (MNP@MDSC) is developed to induce targeted cancer photothermal therapy and coordinate the immunoregulatory properties on the host immune system at the same time.^[^ ^49^ ^]^ This "kill and boost" strategy does not provide training cues for the immune system directly, but equally trigger cascades of adaptive immune responses as vaccines.^[^ ^50^ ^]^

#### 3.1.5 Others

In addition to reagents mentioned above, small‐molecular antagonist, novel biomaterials, etc., can all be applied in CMDNs‐mediated immune intervention.^[^ ^51^ ^]^ For instance, some nucleotide aptamers have antibody‐like functions and can be used to block certain biological processes.^[^ ^52^ ^]^ In another example, the CMDNs platform could be integrated with gene therapy.^[^ ^53^ ^]^ Genetic material could also be introduced into cells in the body by CMDNs to alter the expression of specific immune modulatory genes.^[^ ^54^ ^]^ In Zhuang et al.’s work, a platelet cell membrane‐coated metal–organic framework (MOF) nanodelivery platform for the targeted delivery of siRNA in vivo was developed. High silencing efficiency can be achieved in vitro and in vivo against multiple target genes.

### 3.2 Nanodecoys

**Figure 3:**
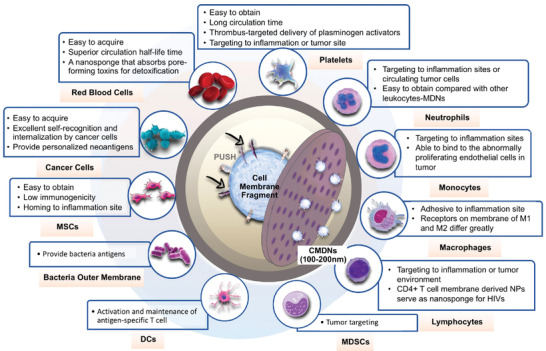
Features of CMDNs from initial source cells. The purified cytomembrane fragments are extruded through polycarbonate film to produce vesicles at nanoscale (100–200 nm). CMDNs possess biological inherencies from the source cells, such as functional surfaces proteins or polysaccharides, which are different from cell to cell. Among these source cells, red blood cells, platelets, cancer cells, and bacteria are relatively easy to prepare and employed as nanocarriers extensively. Guided by adhesion molecules such as selectins and integrins, CMDNs from platelets, leukocytes, and MSCs exhibit substantial affinity to the endothelium of inflammation tissues.

Other than nanocarriers, the unoccupied receptors on cell membrane are able to serve as scavenging sponge to eliminate unwanted viruses, bacteria/toxins, cytokines, chemokines, or growth factors (**Figure** **3**). A notable example for detoxification is erythrosomes, which adsorb pore‐forming toxins to cleanse the body of virulence factors caused by bacterial infections. In another example, a type of T cell mimicking nanoparticles (TCMNPs) are developed to neutralize immunosuppressive components like TGF‐*β*1 and PD‐L1 produced by cancer cells in tumor microenvironment.^[^ ^55^ ^]^ TCMNPs exhibit higher therapeutic efficacy than PD‐L1 blockade in melanoma treatment (≈50% delay). Although in status of underdevelopment, nanodecoy is a promising application of CMDNs for patients to recover from illness especially before the advent of effective drugs or vaccines. For instance, prior to the development of proved vaccines against the COVID‐19, nanodecoy can yet be regarded as a promising strategy to neutralize virus as well as cytokine storm.^[^ ^56^ ^]^

Taken together, instead of developing novel drugs originally, CMDNs leverage the current regulatory approaches to full advantage and boost the existing drugs performance. No matter nanocarriers or nanodecoys, incorporation or depletion, the biomimetic platform successfully integrates the inherit property of cell membrane and the existing immune interventions in a facile way. During the process, some enlightened design strategies are reported and make the synthetic platform more powerful.

## 4 Current Advances on Engineering Strategies of CMDNs

With more efforts on functional CMDNs, the requirements of CMDNs tend to be more specific and detailed. Cytomembrane employed in CMDNs is going through a shift from natural toward modified.^[^ ^57^ ^]^ Meanwhile, the engineering strategies also extend from initial encapsulation to lipid insertion, specific adsorption, membrane fusion, and genetic modification (Figure 3). The flexible set of these engineering strategies has brought to many brilliant CMDNs formulations (**Table** **1**). Such emerging engineering strategies are compatible with each other and any combination is available whenever necessary. In this section, we will provide an overview of current advances on engineering strategies of immune regulatory CMDNs through analyzing some representative studies in turn.

**Table 1:** Examples of CMDNs employed in immune modulation

Name	Year of publication	Disease	Role	Effector cell	Membrane source	Engineering strategy^a)^	Preparation method	Refs.
NP‐R@M‐M	2018	Cancer	Nanocarrier (vaccine)	T cells	Cancer cells	I+II	Mix	[68]
RvD2‐HVs	2019	Cardiovascular cerebrovascular disease	Nanocarrier	Leukocytes	Neutrophils	II	Mix	[14c]
TCPA‐1‐PEV	2020	Pneumonia	Nanocarrier	Inflammatory cells	Platelets	II	Mix	[69]
Nanotoxoid (HLA)	2013	Bacterial infection	Nanocarrier (vaccine)	B cells	RBCs	I+III	Mix	[32b]
PI@EPV	2020	Cancer	Nanocarrier (vaccine)	T cells	OMVs, cancer cells	I+IV	II/gentle stirring with PEG+10% DMSO	[77]
Nano‐Ag@ Erythrosome	2019	Cancer	Nanocarrier (vaccine)	T cells	RBCs, cancer cells	IV	II/sonication	[78]
MOF@FM	2019	Cancer	Nanocarrier (vaccine)	T cells	DCs, cancer cells	I+IV	I/sonication	[80]
VMVs	2015	Viral Infection	Nanocarrier (vaccine)	T, B cells	293T/Hela	V	Genetic engineering	[44b]
Antigen‐loaded OMVs	2021	Cancer	Nanocarrier (vaccine)	T cells	OMVs	V	Genetic engineering	[44a]
[CD80/OVA] NPs	2020	Cancer	Nanocarrier (vaccine)	T cells	Cancer cells	V	Genetic engineering	[81]
1‐MT@PD‐1 NVs	2018	Cancer	Nanocarrier	CD8+T cells	293T	I+V	Genetic engineering/	[83a]
hNVs	2020	Cancer	Nanocarrier & nanodecoy	M*φ*, T cells	Cancer cells, platelets, M1 M*φ*	I+IV+V	Genetic engineering/ II/sonication	[84]
Nanodecoy (COVID‐19)	2020	Viral Infection	Nanocarrier & nanodecoy	‐	293T, monocytes	IV+V	Genetic engineering/ II/sonication	[87]

^a)^I: Encapsulation; II: lipid insertion III: specific adsorption; IV: membrane fusion; V: genetic modification; i: cell–cell fusion; ii: membrane–membrane fusion.

### 4.1 Encapsulation

As the most explored application, cell membrane capsule will potentiate the existing therapeutics, and get rid of severe toxicity with their inherent biocompatibility or specific location‐aggregated property. Encapsulation is a common strategy in CMDNs design because the inner core provides physical support for the membrane, helping to ensure the right‐side‐out membrane protein orientation and performance of the functional units embedded on membrane. Wide range of nanomaterials has been applied for CMDNs, including mesoporous silica nanoparticles,^[^ ^58^ ^]^ polymer,^[^ ^59^ ^]^ iron oxide magnetic nanoparticle,^[^ ^49^, ^60^ ^]^ gelatin nanoparticles,^[^ ^61^ ^]^ MOF,^[^ ^62^ ^]^ gold nanomaterials,^[^ ^63^ ^]^ and upconversion nanoparticles^[^ ^23b^ ^]^ and so on.^[^ ^64^ ^]^ In terms of immune modulation, encapsulation is frequently adopted together with other strategies in CMDNs fabrication. Regarding the monitoring of immune components in the body is based on recognition and interaction, it is more efficient to expose the regulatory components on the membrane rather than encapsulation. Thus, apart from encapsulation, other four approaches are all focused on modification of cell membrane.

### 4.2 Lipid Insertion

Lipid insertion is a method that modifies functional ligands onto cell surface membranes by lipid anchor, which is frequently used in drug‐delivery liposomes.^[^ ^65^ ^]^ Owing to the incorporation process is spontaneous and no direct chemical conjugation is required, lipid insertion is capable of preserving the natural functionality of cell membrane in CMDNs preparation.^[^ ^66^ ^]^ Such engineering strategy provides an unrestricted platform for membrane modification without considering specificity.

**Figure 4:**
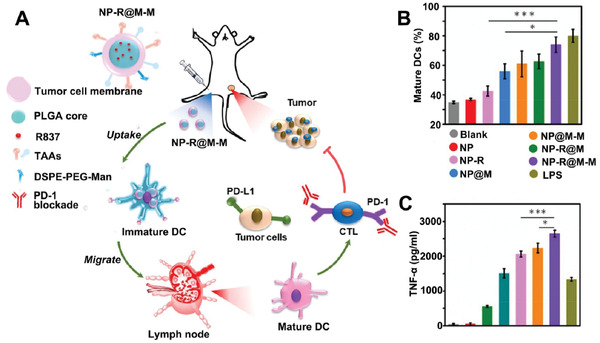
Cell membrane‐coated adjuvant nanoparticles with mannose insertion for anticancer vaccination. A) Schematic of cancer immunotherapy by a combination of engineered [NP‐R@M‐M]NPs and PD‐1 blockade. [NP‐R@M‐M]NPs act as an anticancer vaccine. DSPE‐PEG‐Man insertion for enhanced APC uptake. B,C) Vaccination using NP‐R@M‐M potentiates DCs maturation as well as TNF‐*α* secretion compared with other CMDNs formulations. Mannose modification is an effective way to enhance the potency of vaccines. Reproduced with permission.^[^ ^68^ ^]^ Copyright 2018, American Chemical Society.

Lipid insertion enables achieving specific functions, particularly active targeting. The targeted molecules, peptides, and antibodies can be conjugated with the anchor before inserting into the cell membranes to achieve targeting ability.^[^ ^59^, ^67^ ^]^ In addition to targeted ligands, mannose has been used to anchor onto the cell membrane for specific targeting antigen presenting cells (APCs) to induce immune responses. Yang et al. constructed a synthetic anticancer vaccine by adopting this strategy (**Figure** **4**A).^[^ ^68^ ^]^ R837, a Toll‐like receptor‐7 (TLR‐7) agonist, was first loaded within poly(D, L‐lactide‐*co*‐glycolide) (PLGA) nanoparticles in this approach. Then the NP‐R core was encapsulated by B16‐OVA cell membrane and formed NP‐R@M. To improve the uptake of NP‐R@M, membrane‐coated NPs were mixed with lipids. Here, mannose conjugation is intended to target macrophages and DCs which express mannose receptors abundantly to facilitate the process of antigen presentation. Consistent with the expectations, bone marrow‐derived DCs were boosted to a large extent after mannose modification. In this approach, two immune agonists, R837 and mannose, cooperate to stimulate DC maturation and TNF‐a secretion (Figure 4B,C). R837 was encapsulated in the core while mannose was exposed on the outer membrane of the NP‐R@M‐M. Additionally, cancer cell membrane here not only worked as nanocarriers but also provided a whole array of tumor‐associated antigens (TAAs) to APCs, which is regarded as a distinguishing feature of engineered CMDNs from other conventional anticancer vaccines.

In addition to anchor ligands onto cell membrane, some immunomodulatory drugs are lipid‐soluble and their incorporation into cell membrane is naturally occurring. With the targeting effect of cell membrane, these drugs are easily delivered to endothelium of inflammatory site. To rescue neuroinflammation from ischemic stroke, Dong et al. attempted to address the cellular locations of therapeutic delivery in the brain by leveraging neutrophil membrane‐derived nanovesicles.^[^ ^14c^ ^]^ Resolvin D2‐loaded nanovesicles, RvD2‐HVs, successfully accumulated in inflamed brain vasculature and alleviate inflammation. In another example, Ma et al. made use of engineered platelet‐derived extracellular vesicles to treat pneumonia.^[^ ^69^ ^]^ Pulmonary inflammation and accompanying cytokine storm syndromes were efficiently inhibited.

### 4.3 Specific Adsorption

**Figure 5:**
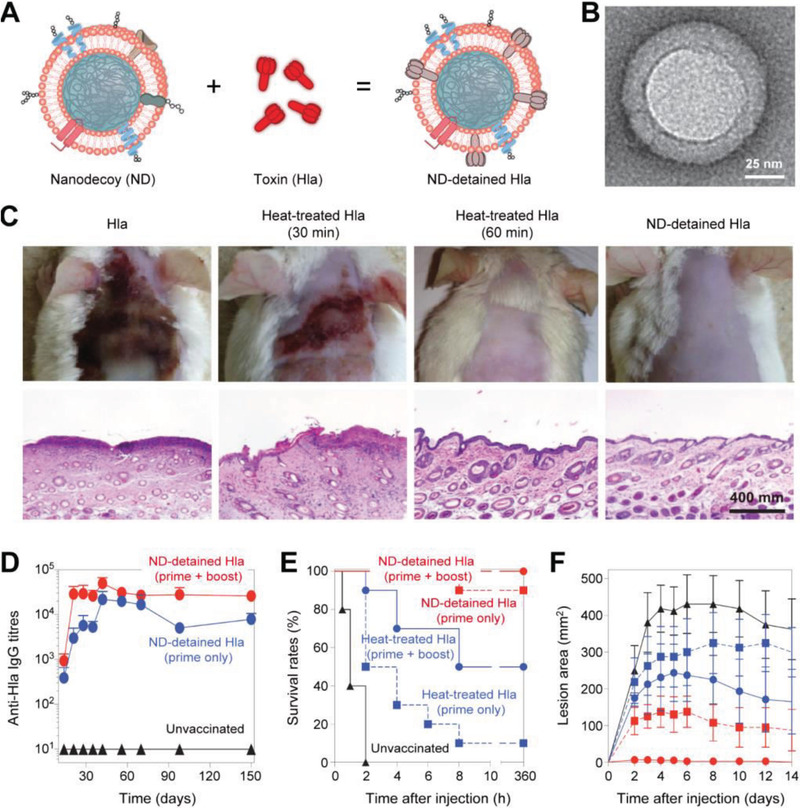
Antibacterial vaccine engineered by PFTs adsorption. A) Bacteria secrete PFTs are capable of causing hemolysis and other damage. PFTs adsorbing RBC membrane‐coated nanoparticles enabling the production of antibodies against PFTs. The resulting antibodies will further protect cells in the body via neutralizing PFTs in circulation. B) TEM image of the particle vectors with uranyl‐acetate staining. C) Images of mice and haematoxylin and eosin (H&E) slices. D) Time course of anti‐Hla IgG titres in unvaccinated mice (black triangles) and mice immunized with ND‐detained Hla (prime + boost; red circles) or ND‐detained Hla (prime only). E) Survival rates of mice following injections of Hla on day 21. F) Comparison of skin lesion size following injections of Hla. Reproduced with permission.^[^ ^70^ ^]^ Copyright 2013, Springer Nature.

Other than unspecific binding, ligand–receptor interaction could also be employed in membrane engineering. The idea of membrane modification via bacteria toxin adsorption has been put to use by Hu et al. for the treatment and prevention of bacterial infection as early as 2013 (**Figure** **5**A,B).^[^ ^32^, ^70^ ^]^ In this approach, nondisrupted staphylococcal *α*‐hemolysin (HLA)‐adsorbed nanoerythrosomes are fabricated as toxoid vaccination against staphylococcal infection. Pore‐forming toxins (PFTs) are bacterial virulence factors that bind host cell receptors, oligomerize, and insert a functional pore into the membrane that may induce osmotic lysis. HLA, a common class of PFTs which named after their lytic activity against red blood cells, are later confirmed cytotoxic toward multiple cells. Such novel vaccines effectively avoided chemical or heat detoxification induced inefficiency in conventional HLA vaccines preparation (Figure 5D‐F). Based on the factor that red blood cell (RBC) membrane possesses specific affinities to multiple bacterial toxins, this nanoerythrosomes‐mediated toxoid formulation could be extended for multiantigenic antibacterial vaccination as well.^[^ ^71^ ^]^

### 4.4 Membrane Fusion

Membrane fusion is a common behavior of cells in intercellular communication via vesicles. This idea of cell membrane fusion in CMDNs engineering is first reported by Dehaini and her colleagues in 2017.^[^ ^72^ ^]^ They fabricated fused membrane by mixing RBCs‐platelets membrane at a 1:1 protein weight ratio under heating conditions. Fused cell membrane gathers both biological properties of the parent membrane which work better than individual one.^[^ ^73^ ^]^ This ingenious strategy has attracted wide attention and multiple attempts have been made by researchers to advance its novel applications.^[^ ^74^ ^]^ For example, CMDNs covered by platelet membrane tend to possess long‐term blood circulation and enhanced tumor accumulation. Cancer stem cells (CSCs) are regarded as important target for tumor therapy due to their extraordinary talent in defusing radiation treatment and chemotherapy. CSCs membrane‐coated nanoparticles exhibit enhanced CSCs targeting ability on account of homogeneity.^[^ ^75^ ^]^ Compared with single platelet CMDNs, platelet‐CSCs CMDNs gain substantial aggregation in tumor with an emphasis on CSCs. And the anticancer drugs loaded within CMDNs elicited better cancer depleting efficacy.

In addition to deliver therapeutics to lesion location more efficiently, the fused membrane can also be used as vaccine for immune stimulation and regulation.^[^ ^30^ ^]^ Scavenger cells within the liver and spleen are responsible for the clearance of pathogens, senescent cells, and antigen presentation as well. Although bacterial OMVs could stimulate the immune components and trigger the production of antitumor cytokines, their vaccination potency is limited due to the antigenic breadth. Tumor cell membranes which provide whole training cues, nevertheless, causing no defensive immune responses in the body. Similar to eukaryotic membranes, OMVs have an identical bilayer structure. As such, platelet extracellular vesicles (PEVs) fused by OMVs and tumor cell membrane combine advantages of both sides and elicits specific immune responses against cancer.^[^ ^76^ ^]^ In another case, Chen et al. reported a type of aging RBCs mimetic nanoerythrosomes as anticancer vaccine by fusing membranes of RBCs and tumor cells.^[^ ^77^ ^]^ In accord with PEVs, the derived nano‐Ag@erythrosomes provide comprehensive tumor‐associated antigens to educate components in our immune system and inhibit recurrence and metastatic spread of the tumor after resection of the primary tumor.^[^ ^78^ ^]^

**Figure 6:**
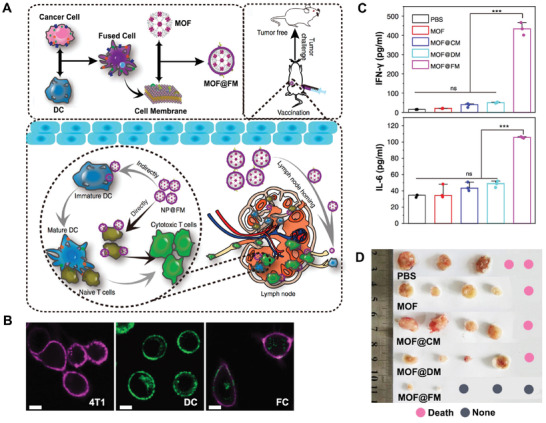
Cell‐free DC vaccine fabricated by DC‐cancer cell fusion. A) Schematic illustration of [Cancer Cell‐DC]NPs for tumor prevention. When subcutaneously delivered into mice, the MOF@FM enhance cytotoxic T cell activity. B) Fusion of DCs and 4T1 cells. 4T1 cells were marked by anti‐CD44‐APC antibody and DCs were labeled with green fluorescence by anti‐MHC II‐FITC antibody. Scale bar = 10 µm. C) Vaccination with MOF@FM, serum IL‐6, and IFN‐*γ* concentration in mice rises to a large extent. D) Tumor occurrence is markedly prevented by MOF@FM compared with other formulations. Healthy mice were immunized twice in every week by subcutaneous injection of MOF@FM and tumor challenge at 7 days after the last immunization. Pink dots represent death of mice. Gray dots represent no tumor is detected. Reproduced with permission.^[^ ^80^ ^]^ Copyright 2019, Springer Nature.

Although the concept of "DC vaccines" has been put forward for a long time, most of the attempts are not successful as exception due to server challenges of complexity in manipulation, inadequate efficacy, and unexpected clinical effects.^[^ ^79^ ^]^ As an improvement, Liu et al. developed engineered CMDNs as cell‐free DC vaccines for tumor prevention (**Figure** **6**A).^[^ ^80^ ^]^ Without introducing exogenous antigens or living cells, this engineered CMDNs here function as conventional DC vaccines with higher controllability and safety. Compared with the previous two approaches, this vaccine starts with primed pseudo‐DCs, bypassing the step of endogenous monocytes/macrophages processing. Unlike the fusion of membrane fragments, membrane fusion in this approach is realized by dendritic cell‐cancer cell fusion. The fused cells were cultured for 6 days and the fused membrane (FM) became homogenous due to the fluidity of cell membrane (Figure 6B). In this process, cancer‐specific epitopes are presented by major histocompatibility complex (MHC). In order to monitor the distribution of these fused CMDNs, a kind of fluorescent MOFs was encapsulated for imaging purpose. It has proven that the MOF@FM nanoparticles possess lymph node homing capacity. Moreover, the "MOF@FM" nanoparticles could be recognized and engulfed by DCs in vivo. Both the two pathways induce DCs‐mediated antigen‐specific T cells and prevent tumor occurrence effectively (Figure 6C,D). Notably, cancer cell membrane in practical could be obtained from patients themselves, coinciding with the concept of personalized nanomedicine. Thus, combining with conventional therapy, the synthetic vaccine with fused membrane will elicit better anticancer potential for both therapy and precaution.

### 4.5 Genetic Modification

Although the fused membrane combines the advantages of parental membranes and shows incomparable excellence, more precise engineering strategy is still in urgent need. Genetic modification has created facile means for membrane engineering, which could obtain new functions by changing the expression of proteins on the cell surface.

**Figure 7:**
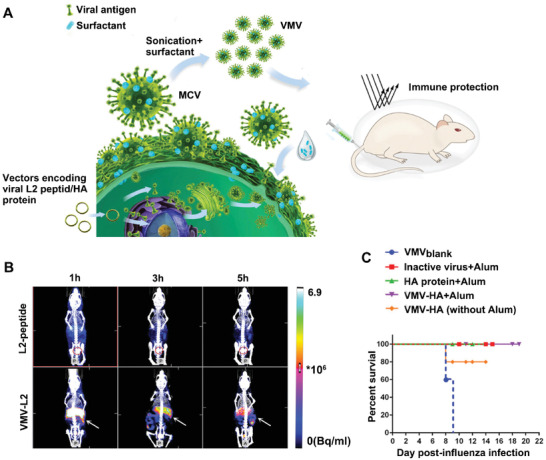
Virus‐mimetic vaccine engineered by genetic modification. A) Schematic of genetically engineered [VMV‐viral antigen]NVs for effective antivirus vaccination. Nucleic acid‐free VMVs with viral antigens enhance adaptive immunity, which ultimately protect mice from viral infection. B) HPV mimetic VMV‐L2 accumulated in mice liver and spleen more effectively than free L2‐peptide. Free L2‐peptide and VMV‐L2 are both labeled by ^99m^Tc (15MBq each mice). SPET/CT images at 1, 3, and 5 h after subcutaneous injection. C) When used to treat H1N1 virus infection, VMV‐HA+Alum elicits equal efficacy to conventional formulations to prolong the life of mice. Mice pretreated with VMV_blank_, inactive virus+Alum, HA protein+Alum, VMV‐HA+Alum, and VMV‐HA (without Alum) separately 4 weeks before challenged with 50 times of lethal dose of mouse adapted H1N1 virus. Reproduced with permission.^[^ ^44b^ ^]^ Copyright 2015, PNAS National Academy of Science.

As mentioned above, engineered capsid L1 subunit of HPV could form VLPs‐mediated vaccines in vitro spontaneously. Similarly, the virus mimetic vaccine could also be presented in an engineered CMDNs way. Zhang et al. developed an effective HPV vaccine via expressing L2 subunit of HPV16 in 293T or Hela cell lines (**Figure** **7**A).^[^ ^44b^ ^]^ To obtain a high yield of displayed 16L2 peptide, a transmembrane signaling peptide derived from integrin is fused in N‐terminal of 16L2 sequence. After treated with different concentrations of sodium deoxycholate in phosphate buffered saline and sonication with 0.05% Triton X‐100, the virus‐mimetic nanovesicles (VMVs) resemble HPV16 in size, shape, and specific immunogenicity. Compared with free L2‐peptide, VMV‐L2 is less susceptible to be cleared and accumulated in liver and spleen more persistently, indicating better vaccination (Figure 7B). Further, this versatile platform was expanded to generate vaccines against H1N1 virus. Primary results showed that the VMV‐HA with Alum adjuvant elicited a comparative protection effect in vivo (Figure 7C). Therefore, as long as the gene sequence encoding specific antigen is available, no matter antigens from pathogenic microbes or tumor‐specific antigens, it means a good chance to generate the corresponding vaccine.

In a recent study, Cheng et al. developed a flexible antigen presentation system via engineered displaying of heterogenous proteins or peptide antigens on the OMV surface.^[^ ^44a^ ^]^ OMVs are nature‐endowed vaccine vectors with inherent adjuvant functionality. Instead of expressing exogenous antigens directly on the surface of OMVs, a "plug‐and‐display" system based on protein tag/protein catcher pairs were introduced in the study to display antigens rapidly and simultaneously. ClyA was selected as the anchor site and the catcher‐fused ClyA was expressed as fusion protein (ClyA‐Catcher, CC) on the OMVs surface. Exogenous antigens which are expected to function with catcher in conjunction with ClyA on the surface of OMVs were tag‐labeled. Hence, this genetic engineering system allows a separate synthesis of CC OMVs and tag‐labeled antigens which may simplify the antigen display process and reduce the production time. Especially for cancer, which has a wide range of neoantigens, the modular design provides a flexible solution for tumor antigen presentation. Overall, genetic modification largely expands the application of bioengineered CMDNs in immune modulation.

**Figure 8:**
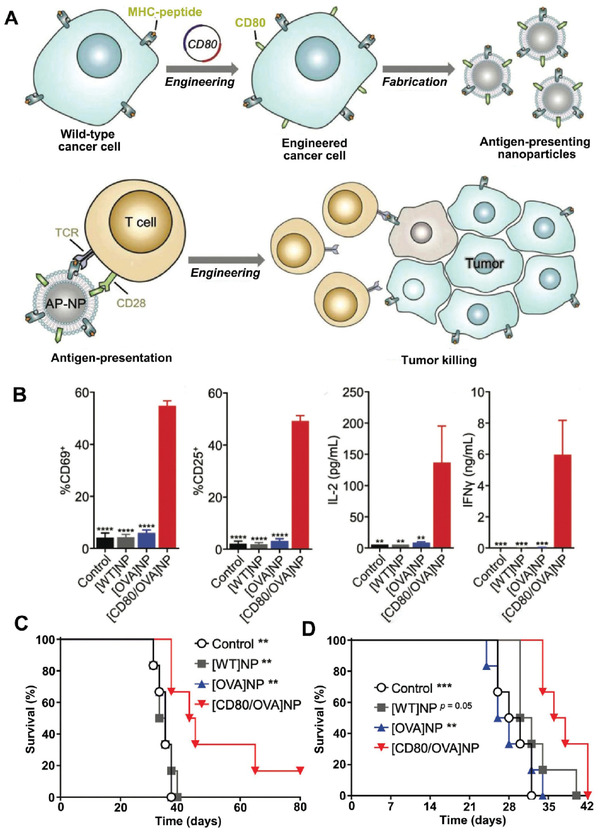
Effective antigen presenting cancer cells with genetic modification. A) Cancer cells are turned into APCs via CD80 and OVA expressed exogenously on them. B) OT‐I CD8+ T cells are boosted by [CD80/OVA]NPs to a large extent. C,D) In vivo prophylactic and therapeutic efficacy of [CD80/OVA]NPs formulation. C,D) When used to vaccinate mice, the [CD80/OVA]NPs can markedly prolong their survival. C) Mice were pretreated with control and the resulting antigen presenting nanoparticles 5 days before tumor cells implanted. D) Mice treated with control and [CD80/OVA]NPs formulation 1 day and 6 days after tumor cells implantation (*n* = 6). Reproduced with permission.^[^ ^81^ ^]^ Copyright 2020, Wiley‐VCH.

Membrane fusion is capable of combing necessary elements together and putting specific vaccination into operation. Sometimes, we can also achieve this purpose through genetic engineering. A prime example is reported by Jiang et al. recently.^[^ ^81^ ^]^ They developed a melanoma cell (B16)‐based pseudo‐APC by CD80 exogenous expression (**Figure** **8**A). CD80, a costimulatory molecule expressed on the surface of APCs, plays a crucial role in T cell activation by delivering costimulatory signals to CD28 on T cells. Here, MHC I originally expressed on cancer cells is leveraged to present tumor‐associated antigens. Because there are ample cytokines necessary for T cell activation in vivo, additional CD80 together with a peptide–MHC complex are generally sufficient to prime T cells. Similar to pseudo‐DC vaccine mentioned above, vesicles derived from the artificial‐APC could act as cell‐free APC vaccine as well. To further help to facilitate the study, a cytosolic form of ovalbumin (OVA) was co‐transfected in the artificial‐APCs. The proportion of activated CD8+T cells increased significantly with [CD80/OVA]NPs treatment compared to [WT‐B16]NPs or [OVA]NPs (Figure 8B). Owing to complexity of cancer, the efficacy of tumor therapy is often not as obvious as recurrence prevention. In vivo results of [CD80/OVA]NPs showed the same tendency (Figure 8C,D). The fact suggests that the emphasis of CMDNs design should be discriminating in dealing with existing tumor or recurrence prevention. Besides vaccines, genetic engineering has been done a great deal to help to deliver aggressive projects.^[^ ^82^ ^]^ As an example, Zhang et al. developed PD‐1 presenting nanovesicles to block massive PD‐L1 on tumor cells.^[^ ^83^ ^]^ Also transgenic therapy, such cell‐free formulations listed here introduces no artificial vector and will undoubtedly improve the safety and controllability of clinical transformation.

### 4.6 Multiple Membrane Engineering

**Figure 9:**
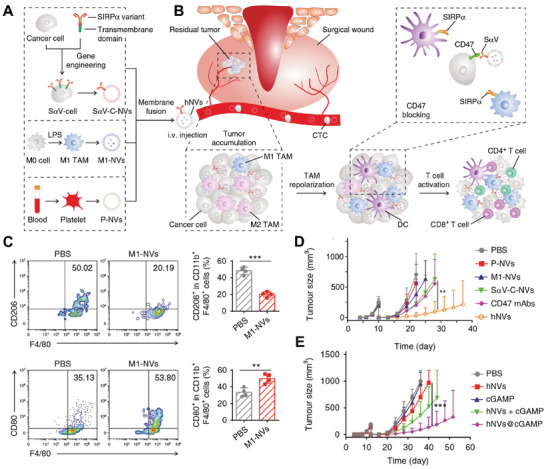
Anticancer CMDNs fabricated by a combing strategy. A) Anticancer hNVs fabricated by SIPR*α* variant‐cancer cell nanovesicles (S*α*V‐C‐NVs), M1 TAM nanovesicles (M1‐NVs), and platelet nanovesicles (P‐NVs). B) Schematic illustration of the main mechanisms of hNVs to inhibit tumor recurrence and metastasis, including post‐surgical tumor bed accumulation, CD47‐SIPR*α* pathway blocking, CTCs interaction, TAMs toward M1 polarization, antitumor T cell activation. C) M1‐NVs cause TAM repolarization toward M1 macrophage with in B16F10 subcutaneous burden. D,E) Tumor recurrence was significantly suppressed by hNVs, especially in hNVs with cGAMP encapsulation. Reproduced with permission.^[^ ^84^ ^]^ Copyright 2020, Springer Nature.

Monotherapy often fails to meet exceptions in dealing with complex diseases, such as cancer. Rao et al. constructed a type of multitargets hybrid nanovesicles (hNVs) to inhibit the recurrence and metastasis of malignant tumor.^[^ ^84^ ^]^ Three engineering strategies are adopted in this approach, including genetic modification, membrane fusion, and drug encapsulation (**Figure** **9**A,B). First, an SIPR*α* variant (S*α*V) which showed superior affinity to CD47 was engineered on the surface of cancer cells.^[^ ^85^ ^]^ And then, three kind of vesicles are fused, including SIPR*α* variant‐cancer cell nanovesicles (S*α*V‐C‐NVs), M1 TAM nanovesicles (M1‐NVs), and platelet nanovesicles (P‐NVs). Among them, S*α*V‐C‐NVs are designed to block CD47‐SIPR*α* pathway to improve the sensitivity of phagocytes, M1‐NVs are used to repolarize M2‐to‐M1 TAMs, and P‐NVs are mainly employed to enhance affinity to post‐surgical tumor bed, as well as circulating tumor cells (CTCs) (Figure 9C). In vivo results indicated that hNVs produce better activity to reduce tumor relapse compared with three nanovesicles engineered before membrane fusion (Figure 9D). Further, by loading cGAMP, a STING agonist, hNVs@cGAMP elicited more efficient inhibition of tumor recurrence (Figure 9E). Together, this scalable CMDNs platform provided more adaptable ways for the fabrication of multifunctional CMDNs.

**Figure 10:**
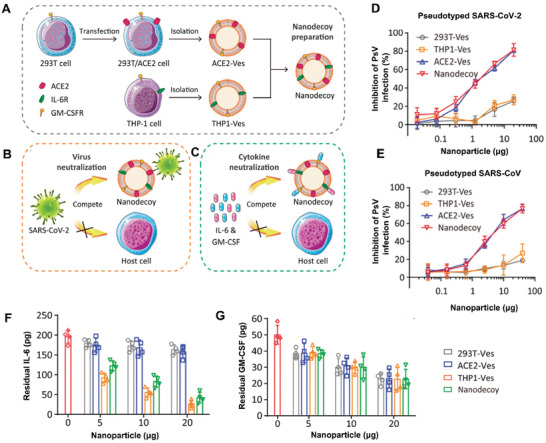
Engineered CMDNs as nanodecoy against COVID‐19 infection. A) Nanodecoy against COVID‐19 with an appreciable amount of ACE2 and receptors of inflammatory factors is produced by genetic modification and membrane fusion. B,C) The nanodecoy is functionalized to adsorb coronavirus as well as excessive inflammatory factors. D,E) The deriving nanodecoy is able to protect cells from pseudotyped SARS‐CoV‐2 and SARS‐CoV infection markedly. F,G) IL‐6 and GM‐CSF are neutralized by the nanodecoy to a large extent. Reproduced with permission.^[^ ^87^ ^]^ Copyright 2020, PNAS National Academy of Science.

It is notable that engineering strategies mentioned above are not only available for nanocarriers to introduce immune modulatory components but also for nanodecoys to remove unwanted harmful substances.^[^ ^86^ ^]^ Rao et al. developed decoying engineered CMDNs for COVID‐19 which were engineered by a two‐step strategy.^[^ ^87^ ^]^ The first step is genetic modification (**Figure** **10**A). Due to the fact that spike protein angiotensin converting enzyme II (ACE2) plays an essential role for SARS‐CoV‐2 infection, the S1 subunit of ACE2 was genetically expressed in 293T cells.^[^ ^88^ ^]^ The coronavirus was entrapped by the derived ACE2 vesicles and lack of potency to infect pulmonary epithelial cells (Figure 10B,D,E). To dealing with cytokine storm syndrome most COVID‐19 patients experiencing, herein, nanovesicles derived from human myeloid mononuclear THP1 cells with abundant cytokine receptors were leveraged as sponges to deplete the extensive cytokines. The second step of this approach is membrane fusion of ACE2‐293T and THP1 (Figure 10C,F,G). As a result, the hybrid nanodecoys provide an effective protection against coronavirus and inflammatory factors at the same time. This strategy is of high clinical transformation value in responding to sudden pandemic especially prior to the effective therapeutics are available.

Engineered CMDNs have broken up the original restriction of natural membrane, with increased efficiency in therapeutics delivery or harmful substances depletion. Altogether, the most eye‐catching advantages for engineered CMDNs are listed below. 1) Personalization: cancer has become a worldwide medical problem largely due to the heterogeneity of cancer cells as well as individual patients. Only vaccine which presents antigens reflecting patient's own condition is worthy of a "personalized vaccine." Engineered CMDNs‐mediated anticancer vaccines which are ready to provide whole array of training cues to our immune system are personalized vaccines in real sense. 2) Controllability and safety: compared with cellular therapy at present, the cell‐free formulation of engineered CMDNs is more feasible and controllable. Besides, it is not necessary to worry about the carcinogenicity of the genetically modified cells employed in cellular therapy. Besides, membrane from autogenic cells will minimize the risk of additional immunological rejection or infectious diseases transmit from person to person. Thus, engineered CMDNs possess a higher safety coefficient. 3) Flexibility: engineered CMDNs platform is of great scalability attributed to the adaptability of cell membrane and diversity of reagents CMDNs carried. In real practice, the formulation could be adjusted according to disease own characteristics and regulation rule of our immune system.

## 5 Challenges before Clinical Translation

**Figure 11:**
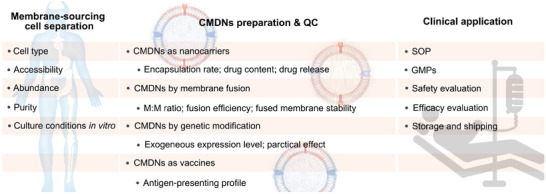
Problems to be solved in CMDNs’ clinical applications. For the initial stage of CMDNs, there is a lot of work to be done in clinical application. We have made a short list of certain problems need to be further studied in chronological order of CMDNs’ development, which is roughly divided into three steps: membrane sourcing‐cell separation, CMDNs preparation & QC, and clinical application.

Even though cellular therapy and nanoparticle‐delivery systems have achieved a series of successes, the clinical application of CMDNs as emerging personalized immune modulators is still challenging. Herein, some problems demanding prompt solution for CMDNs are summed up to reach their extent practical application (**Figure** **11**). Membrane‐sourcing cell selection is one crucial issue in CMDNs translation. According to the application purpose of CMDNs, function matching cells are primarily locked. Besides, some feasibility related issues like accessibility, abundance, purification method, and culture conditions in vitro of the candidate cells should be taken into consideration comprehensively. For example, DCs are of low content but high heterogeneity, hence, large numbers of DCs can be generated in vitro from monocytes using several differentiation and maturation protocols.^[^ ^89^ ^]^

Another vital link in the process is CMDNs preparation. Reagents encapsulation is employed in most design of CMDNs. Hence, the remaining problems will be mentioned again, including encapsulation rate, drug content, and drug release. As for fused membrane‐mediated CMDNs, many points are worth focusing on, like M:M ratio, fusion efficiency, and fused membrane stability. To CMDNs prepared by genetic modification, exogenous expression level and practical effect of the exogenous component are all crucial elements in determining the final performance of CMDNs in immune modulation. In particular, to engineered CMDNs as anticancer vaccines, identification of TAA‐presenting profile and the evaluation system should be established in detail. Overall, each step in the process of CMDNs preparation requires strictly quality control (QC).

Similar to patent drugs, establishment of industrial standard for CMDNs as personalized nanomedicine is necessary and will be certainly beneficial to their good development. For example, standard operation procedure, good manufacturing practices certification, standard safety, and efficacy evaluation system and so on. Although CMDNs have a long way to go before clinical transformation, they still belong to the most boosting branch of personalized nanomedicine.

## 6 Perspectives and Outlook

**Figure 12:**
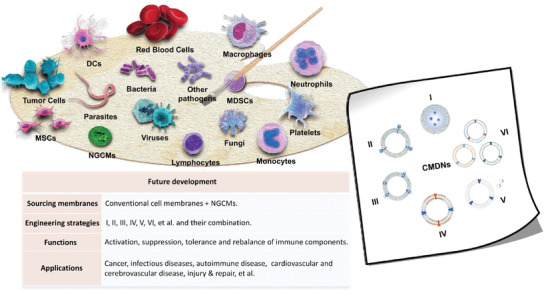
Future prospects of CMDNs for immune modulation. Other than conventional cell membranes, there has to be NGCMs which are universally available among different patients in the future. Such ready‐to‐use cell membranes are conductive to quality control of CMDNs and more efficient clinical application. Further, we have proposed a hybridization made of a cocktail of CMDNs (VI) rather than membrane fusion, which could be regarded as the sixth basic engineering strategies of CMDNs (I: encapsulation; II: lipid insertion; III: specific adsorption; IV: membrane fusion; V: genetic modification). Additionally, immune function of CMDNs is not limited to activation or suppression. Immune regulatory CMDNs are bound to play bigger roles in reestablishing the harmony of immune components, helping to cure more diseases.

In general, CMDNs are at the beginning stage, hence, their design and application are still under exploration. With the progresses on the relationship between immune dysregulation and disease development, more spaces would be expected for CMDNs in immune modulation. Next, we will discuss the practical application of CMDNs from three aspects, including membrane sources, engineering strategy, and novel applications of CMDNs in immune regulation (**Figure** **12**).

### 6.1 Membrane Sources

Cell membrane‐derived nanoplatform is a cytomembrane‐based field, hence, the sourcing membrane selection has always been an essential issue. The patient‐specific nature has shown incomparable benefits, nevertheless, it also means labor‐intensive work and increased treatment production time. In the progression of parallel cellular therapy, a number of research institutions and pharmaceutical companies are promoting the development of the next‐generation CAR‐T therapy.^[^ ^90^ ^]^ It is an allogenic CAR‐T approach causing no obvious rejection or significant graft‐versus‐host disease.^[^ ^91^ ^]^ It is notable that CMDNs will go through a similar revolution to be "off‐the‐shelf" ones. Next‐generation cell membranes (NGCMs) which enable cross‐application among patients will greatly improve the efficiency of clinical application.

### 6.2 Engineering Strategies

If cell membrane forms the substance of CMDNs, then the engineering process is reshaping of the platform. Encapsulation, lipid insertion, specific adsorption, membrane fusion, and genetic modification are five basic means employed in CMDNs fabrication. Each strategy can be adopted separately or combine with other ones at will. With the deep‐going of the study, novel engineering strategies will get added. For example, the concept of hybrid membrane is unnecessary to narrow to membrane fusion. A cocktail of distinct CMDNs is also feasible. In some severe viral infections, many pathogenic factors will cause damage to the body, such as virus, associated cytokine storm, and autoantibodies. To trap these key pathogenic factors more efficiently, the candidate membrane could be modified by expressing designated receptors genetically to achieve differential adsorption. Instead of all together in one cell, the designated receptors could be expressed in candidate cells separately. The final formulation is a cocktail of several kinds of nanovesicles. This method not only reduces the difficulty of multigene modification, but attempts to bypass the problem of low success rate of multi‐membrane fusion. Moreover, novel techniques and exploration of disease will pioneer the development of CMDNs. Electro‐transformation or transposon‐mediated transfection, for instance, will improve the safety and efficacy of genetic engineering to a large extent.^[^ ^92^ ^]^ Therefore, with the new techniques and new findings, engineering strategies will change the landscape of CMDNs.

### 6.3 Immunomodulatory Applications

Current applications of engineered CMDNs in immune modulation mainly focus on vaccine against cancer and infectious diseases. A few random cases of cardiovascular or autoimmune diseases are all based on the function of drugs themselves. Along with the findings about the crosslink between immune disorders and disease progression, immune regulatory CMDNs will play a bigger role in diseases alleviation.^[^ ^11a^ ^]^ Our immune system is a built‐in system of checks and balances. It often displays as unity of two or more opposites, such as Th1/Th2/Th17/Treg paradigm of CD4+ T cells or M1/M2 polarization of macrophages.^[^ ^93^ ^]^ Many diseases, including autoimmune diseases, infectious diseases, inflammation, cancer, vascular diseases, and nerves system diseases, are accompanied with deviation of the existing balance. And the purpose of immune intervention is to restore the harmony of subfractions through precise coordination. The moderate components could be reboosted by stimuli like vaccines. And the hypoactive ones could be weakened by inhibitory modulators, or rebalanced by propping up the opposites. The study of hybrid nanovesicles reported by Rao et al. is a prime example to promote the M2‐to‐M1 M*φ* repolarization.^[^ ^84^ ^]^ Other than cancer progression, macrophage M1/M2 polarization have been demonstrated to be involved in many pathological processes.^[^ ^94^ ^]^ And Th1/Th2/Th17/Treg paradigm is closely related to most autoimmune diseases.^[^ ^95^ ^]^ Hence, the imbalanced paradigm is a good break point for engineered CMDNs to develop new applications.

## 7 Conclusions

The above step by step, is a systematic overview on the progress of engineered CMDNs in immune modulation. Starting with the molecular basis of cell membrane function, immune regulatory roles and basic engineering strategies of CMDNs are discussed. A considerable length of this review is devoted to the analysis of some representative cases, with an emphasis on the logic of CMDNs design. No matter bringing about therapeutic or taking away harmful components, the most essential role of the bilayer membrane in CMDNs is vehicle. Versatile engineering process breaks the limitation of natural membrane, creating infinite possibilities for the application of CMDNs. Especially as anticancer vaccine, a lot of engineered CMDNs are able to provide whole array of patient‐specific TAAs, realizing personalized vaccination. Moreover, inspired by the success of cellular immunotherapy, engineered CMDNs‐mediated cell‐free therapy holds greater promise for clinical translation. Immunomodulatory CMDNs are still in their infancy. From acquisition of the membrane‐sourcing cells to the certification for human‐body application, an immense amount of work remains. Each segment in design, fabrication, and clinical translation can be further leveraged. With versatile membrane sources, infinite modification strategies, continuous innovation of theory and technology, the biomimetic engineered CMDNs platform is definitely to provide better solutions to the pressing medical problems.

## Conflict of Interest

The authors declare no conflict of interest.
